# Prevalence of *Entamoeba* species in captive primates in zoological gardens in the UK

**DOI:** 10.7717/peerj.492

**Published:** 2014-07-29

**Authors:** Carl S. Regan, Lisa Yon, Maqsud Hossain, Hany M. Elsheikha

**Affiliations:** School of Veterinary Medicine and Science, University of Nottingham, Loughborough, Leicestershire, UK

**Keywords:** *Entamoeba*, *Homo sapiens*, Zoonosis, Public health, Phylogenetics, Prevalence, Zoos, Nonhuman primates

## Abstract

The aim of this study was to determine the prevalence of amoebic infection in non-human primates (NHPs) from six Zoological gardens in the United Kingdom. Initially, 126 faecal samples were collected from 37 individually identified NHPs at Twycross Zoo, UK, and were subjected to microscopic examination. A subsequent, nationwide experiment included 350 faecal samples from 89 individually identified NHPs and 73 unidentified NHPs from a number of UK captive wildlife facilities: Twycross Zoo (*n* = 60), Colchester Zoo (*n* = 3), Edinburgh Zoo (*n* = 6), Port Lympne Wild Animal Park (*n* = 58), Howletts Wild Animal Park (*n* = 31), and Cotswold Wildlife Park (*n* = 4). Samples were examined by PCR and sequencing using four specific primer sets designed to differentiate between the pathogenic *E. histolytica*, the non-pathogenic *E. dispar*, and non-pathogenic uninucleate cyst-producing *Entamoeba* species. In the first experiment, *Entamoeba* was detected in 30 primates (81.1%). Six (16.2%) primates were infected with *E. histolytica* species complex. The highest carriage of *Entamoeba* species was found in Old World Colobinae primates. In the nationwide experiment, molecular analysis of faecal samples revealed notable rates of *Entamoeba* infection (101 samples, 28.9%), including one sample infected with *E. histolytica*, 14 samples with *E. dispar*, and 86 samples with uninucleated-cyst producing *Entamoeba* species. Sequences of positive uninucleated-cyst producing *Entamoeba* samples from Twycross Zoo clustered with the *E. polecki* reference sequences ST4 reported in *Homo sapiens*, and are widely separated from other *Entamoeba* species. These findings suggest a low prevalence of the pathogenic *Entamoeba* infection, but notable prevalence of non-pathogenic *E. polecki* infection in NHPs in the UK.

## Introduction

*Entamoeba* (family Entamoebidae) is a genus of diverse intestinal protists found in humans, nonhuman primates (NHPs) and other animals. It encompasses several species, including *E. histolytica*, *E. dispar*, *E. moshkovskii*, *E. polecki*, *E. nutalli*, *E. chattoni*, *E. coli*, *E. hartmanni*, *E. ecuadoriensis* and *E. Bangladeshi*. NHPs harbour a number of *Entamoeba* spp. of varied importance to human and domestic animal health. *E. histolytica* species complex (*E. histolytica*, *E. dispar* and *E. moshkovskii*) are morphologically indistinguishable, but have different virulence capabilities. *E. histolytica* is the most important zoonotic pathogen (Sargeaunt, Williams & Jones, 1982; Verweij et al., 2003; Ekanayake et al., 2006), and has been reported in NHPs, causing intra- and extra-intestinal disease (Solaymani-Mohammadi et al., 2006; Ulrich et al., 2010). Also, *E. histolytica* is known to be responsible for 50 million human cases of haemorrhagic colitis and extra-intestinal abcessation, and 100,000 deaths annually (World Health Organization, 1997). In contrast, *E. dispar* is able to colonize the intestine, but is noninvasive. *E. moshkovskii* is primarily free-living and the ability to cause disease in human is still unclear (Heredia, Fonseca & Lopez, 2012). Also, human diseases linked to the uninucleated cyst-producing *E. chattoni* have been attributed to contact with monkeys (Sargeaunt, Patrick & O’Keeffe, 1992). *E. nuttalli* and *E. histolytica* species complex have previously been confused or misidentified on routine examination due to their morphological similarity, but are now considered separate species with restricted host specificity (Tachibana et al., 2013).

Microscopic examination of faecal samples has been traditionally the primary method of *Entamoeba* detection; however, it does not allow the differentiation of the pathogenic *E. histolytica* from the non-pathogenic *Entamoeba* spp (Kebede et al., 2003; Verweij et al., 2003; Fotedar et al., 2007). Knowledge of *Entamoeba* epidemiology and evolution has considerably progressed in recent years, with improved isolation, identification, and genotyping methods (Levecke et al., 2010; Stensvold et al., 2011). These molecular methods have detected considerable diversity within the genus, and enabled the detection and distinction of species (including the so-called ribosomal lineages) that cannot be differentiated by traditional parasitological methods. Despite the continued importance of *Entamoeba* spp and the known susceptibility of NHPs to infection very little information is available on the prevalence of *Entamoeba* infection in NHP populations in the United Kingdom. Given its zoonotic potential and public health impact, including in a zoological setting the present study assessed and compared *Entamoeba* prevalence in captive primates in various zoological gardens throughout the United Kingdom using molecular methods of *Entamoeba* detection.

## Materials & methods

### Study areas and sampling design

A preliminary study was performed to establish the prevalence of *Entamoeba* infection in primates in a single zoological park in the United Kingdom, and to identify which families of primates required the most focus during the subsequent nationwide study. Hence, Twycross Zoo was chosen as the preliminary study site as it houses a wide variety of primate species and families, and amoebic infection had been identified historically and was suspected in their primates at the time of the study. Thirty-seven primates were available for inclusion within the study, including six species of primates from 23 enclosures (Table 1). To identify individual primates in group enclosures a feed item for each primate was impregnated with approximately 0.5 g of different coloured edible cake glitter (Rainbow Dust Colours Limited, Lockstock Hall Preston, England) and fed during the morning by the keeper. Each group individual was assigned and fed a different glitter colour from two days before sample collection until successful completion of sample collection; this was typically two to five days. All stool samples in each enclosure were collected separately on clean disposable paper plates during morning cleaning by keepers until three stools per primate were identified from the glitter colour allocated for each primate. Three samples per primate were collected to account for intermittently shed *Entamoeba*. A representative 3–5 g stool sample for each animal (identified by different glitter colours) was placed into a labeled clean 7 ml plastic bijoux, using a clean wooden swab stick, containing 10% formalin, and was stored at 5 ° C until further processing. Age, sex, species, treatment with amoebicidal medication and enclosure identity were recorded.

**Table 1:** The prevalence of *Entamoeba* spp. in non-human primates from Twycross Zoo.

Species	Family	*E. histolytica complex*	*E. hartmanni*	*E. coli*
Black Howler Monkey (*Alouatta caraya*; *n* = 17)	Atelidae	11.8% (2)	35.3% (6)	70.6% (12)
Brown Woolley Monkey (*Lagothrix lagotricha*; *n* = 6)	Atelidae	16.7% (1)	66.7% (4)	66.7% (4)
Eastern Javan Langur (*Trachypithecus auratus auratus*; *n* = 9)	Colobinae	22.2% (2)	88.9% (8)	77.8% (7)
Dusky Leaf Monkey (*Trachypithecus obscures*; *n* = 1)	Colobinae	100% (1)	100% (1)	0%
Golden Lion Tamarin (*Leontopithecus rosalia*; *n* = 2)	Callitrichidae	0%	0%	0%
Golden-headed Lion Tamarin (*Leontopithecus chrysomelas*; *n* = 2)	Callitrichidae	0%	0%	0%
**Total** (***n*** = **37**)		**16.2% (6)**	**51.4% (19)**	**62.2% (23)**

**Figure 1:**
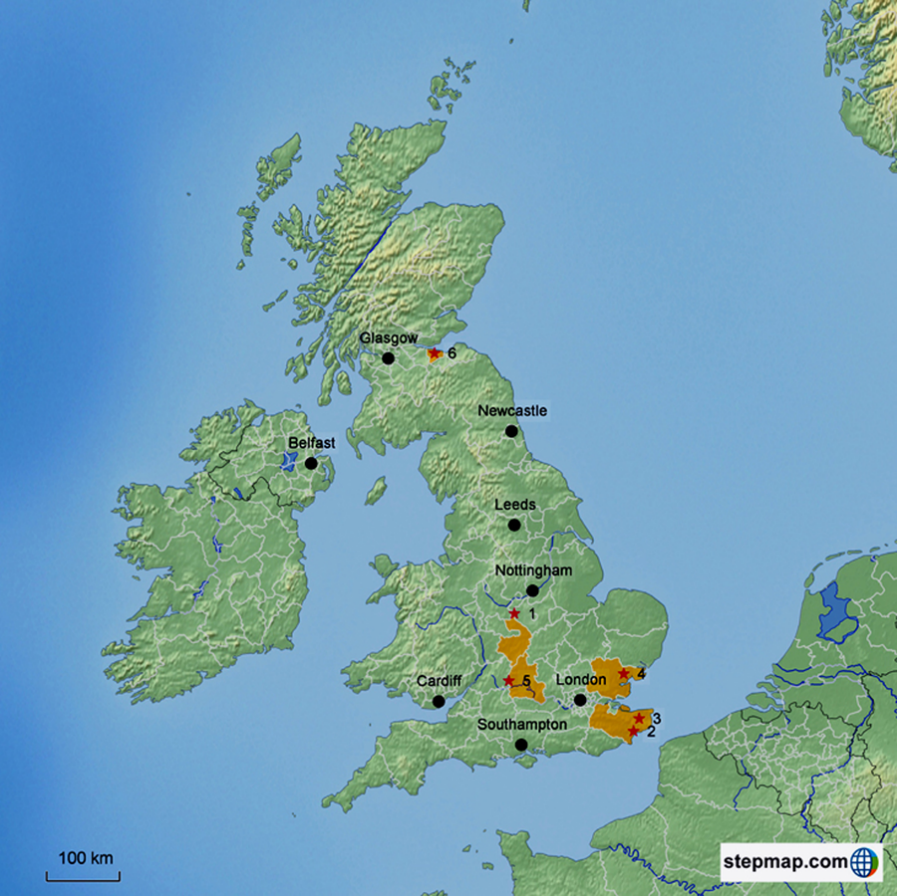
Map of The United Kingdom showing the sampling locations. Six zoological gardens are indicated by red solid stars. The map was created by using the STEP MAP web tool. ^1^ Twycross Zoo, Atherstone, Midlands, CV9 3PX, England. ^2^ Port Lympne Wild Animal Park, Lympne, Hythe, Kent, CT21 4LR, England. ^3^ Howletts Wild Animal Park, Bridge, Canterbury, CT4 65AE, England. ^4^ Colchester Zoo, Stanway, Colchester, Essex, CO3 0SL, England. ^5^ Cotswold Wildlife Park, Bradwell Grove, Burford, Oxfordshire, OX18 4JP, England. ^6^ Edinburgh Zoo, Edinburgh, City of Edinburgh, E12 6TS, Scotland.

Subsequent to the preliminary study, a nationwide epidemiological study was conducted to identify the prevalence of *Entamoeba* infection, *E. histolytica*, *E. dispar* and uninucleate cyst-producing species, within Colobinae primates at six different zoos in the United Kingdom (Fig. 1). Primates from the Colobinae family (genus *Semnopithecus*, *Trachypithecus* or *Presbytis*) were selected as the sample population for the nationwide study based on two reasons. Firstly, the preliminary data from Twycross Zoo demonstrated the highest prevalence of amoebiasis in the Old World Colobinae monkeys. This is in agreement with results from other studies (Tachibana et al., 2001; Levecke et al., 2007). Secondly, primates from the Colobinae family have specialised sacculated stomachs, an adaption to their leaf-eating lifestyle, which provides favorable conditions for ingested *Entamoeba* cyst excystation, and trophozoite tissue invasion (Mätz-Rensing et al., 2004; Ulrich et al., 2010).

A total 350 samples were collected from 162 primates from six zoological parks within the United Kingdom (Table 2), between July 2010 and August 2011. This sample group included primarily primates from the Colobinae family, but also some New World monkeys. All zoological parks housed primates and non-primate species. Primates occupied indoor concrete enclosures with access to external grassed sections. Same species primates occupied mixed sex group enclosures. Some primates were housed alone for medical reasons, or due to social incompatibility with the rest of the group. It was possible to collect repeat samples from four primates sampled in the preliminary study; all other primates from Twycross Zoo were unavailable for sampling. The same stool collection technique was used as for the preliminary study with one modification to facilitate molecular examination of samples: stools were collected into 70% ethanol, not 10% formalin. The primate keepers at each facility administered the glitter and collected the samples. Two hundred and seventy-four stool samples could be associated with 89 individually identified primates; however, some stool samples collected could not be attributed to a specific primate from within a group enclosure. This was due to the limitations of deciphering different glitter colours when dealing with large number of primates, and hence glitter colours, in one enclosure. Hence, 76 stools from the remaining 73 primates had to be collated as samples from eleven groups of NHPs (Table 2). The entirety of each stool sample was examined grossly for the presence of blood as a possible indication of gastrointestinal illness and potential parasitism. Thirty two (82.1%) of primates had been treated with a vitamin D3 supplement and 10 day course of metronidazole (Flagyl) followed by 10 days of diloxinide furoate in the six months prior to sample collection.

**Table 2:** Non-human primates sampled in the nationwide study.

Study site	Species of primate	Number of primates sampled	
		Individually identified	Unidentified primates (no. of group)
Twycross Zoo	Eastern Javan Langur (*Trachypithecus auratus auratus*)	9	4 (1)
	Black Howler Monkey (*Alouatta caraya*)	15	11 (2)
	Woolley Monkeys (*Lagothrix lagotricha*)	6	–
	Dusky Leaf Monkey (*Trachypithecus obscures*)	1	–
	Golden Lion Tamarin (*Leontopithecus rosalia*)	2	–
	Golden-headed Lion Tamarin (*Leontopithecus chrysomelas*)	2	–
	Francois Langur (*Trachypithecus francoisi*)	5	–
	Dusky Leaf Monkey (*Trachypithecus obscures*)	5	–
Port Lympne Wild Animal Park	Eastern Javan Langur (*Trachypithecus auratus auratus*)	9	39 (4)
	Grizzled Leaf Monkey (*Presbytis comata*)	–	7 (1)
	Banded Leaf Monkey (*Presbytis femoralis*)	–	3 (1)
Howletts Wild Animal Park	Banded Leaf Monkey (*Presbytis femoralis*)	5	–
	Dusky Leaf Monkey (*Trachypithecus obscures*)	14	–
	Francois Langur (*Trachypithecus francoisi*)	2	–
	Grizzled Leaf Monkey (*Presbytis comata*)	8	–
	Eastern Javan Langur (*Trachypithecus auratus auratus*)	2	–
Colchester Zoo	Silvery Langur (*Trachypithecus cristatus cristatus*)	3	–
Cotswold Wildlife Park	Purple-faced Langur (*Trachypithecus vetulus monticola*)	1	3 (1)
Edinburgh Zoo	Purple-faced Langur (*Trachypithecus vetulus vetulus*)	–	6 (1)
**Total**		**89**	**73 (11)**

The study was approved by The University of Nottingham (UK) School of Veterinary Medicine and Science (SVMS) Ethical Review Committee. The Committee reviews all research studies involving School personnel and is chaired by Professor David Haig. The committee passed this study as good to proceed, not requiring any further ethical review.

### Parasite identification

All formalin preserved samples were analysed microscopically using a modified Ridley’s formol-ether concentration technique, which enhances microscopic sensitivity by producing ‘cleaner’ samples that are more efficient to examine. Following sedimentation, samples were then examined microscopically for the presence of *Entamoeba* species from the *E. histolytica* complex (*E. histolytica*, *E. dispar* and *E. moshkovskii*), *E. coli* and *E. hartmanni*. Data was analyzed using Minitab 15. Binary logistic regression was used to demonstrate statistical significance between prevalence of infection and primate demographics. All prevalence data is derived using the total number of primates as the denominator.

### Molecular analyses

QIAamp DNA Stool Mini Kit (QIAgen, UK) was used according to the manufacturer’s instructions to extract parasite DNA directly from faeces. Technique modifications to improve the yield and purity of DNA extracts included increasing the lysis temperature to 95 ° C and adding an extra wash prior to sample elution with Buffer AE. Concentration and DNA purity in sample extracts was analyzed, using a Thermo Scientific NanoDrop™1000 Spectrophotometer, prior to PCR amplification. The strategy used for selection of PCR primers (Table 3) was based on the use of previously published diagnostic primers for the mononucleate *Entamoeba* species, *E. histolytica* and *E. dispar* (Ali, Zaki & Clark, 2005). Both species have been previously found in the faeces of NHPs. It is important to discriminate *E. histolytica* from other nonpathogenic amoebas because *E. histolytica* carries the risk of zoonosis (Rivera & Kanbara, 1999; Tachibana et al., 2000; Tachibana et al., 2001; Verweij et al., 2003; Rivera, Yason & Adao, 2010; Feng et al., 2011). Also, we used two species complex specific primers to amplify uninucleate cyst-producing species, but not tetra- or octonucleate cyst-producing species. All PCR products were subjected to DNA sequencing to identify the species/subtype of each amplicon including those amplified by the species diagnostic primers.

**Table 3:** Primer sets and PCR conditions used in the present study.

	Forward primer	Reverse primer	Amplification reaction
Primer set 2^*^	Primer 5.1: (5″-AAG GAT AAC TCT TGT TAA TTG CAG-3″)	Primer 3.2: (5″-TGT CTA AAT TAC CCC AAT TTC C-3″)	30 cycles of 94 ° C, 57 ° C, and 72 ° C each for 30 s, followed by a final 2 min at 72 ° C
Primer set 3^*^	Primer 5.2: (5″-GGA ATA GCT TTT TGA GAA GAA GG-3″)	Primer 3.2: (5″-TGT CTA AAT TAC CCC AAT TTC C-3″)	30 cycles of 94 ° C, 57 ° C, and 72 ° C each for 30 s, followed by a final 2 min at 72 ° C
*E. histolytica*			
	RRH5: (5″-GCG CCT TTT TAT TCA ATA TAC TCC-3″)	RRH3: (5″-GGA TGA AGA TAT CTT CAC AGG G-3″)	30 cycles of 94 ° C, 59 ° C , and 72 ° C each for 30 s, followed by a final 2 min at 72 ° C
*E. dispar*			
	RRD5: (5″-CAT GAG GCG CCT TTT TAT CA-3″)	RRD3: (5″-AGG GGA TGA TGA TAT TGA ACA CAC TC-3″)	30 cycles of 94 ° C, 59 ° C , and 72 ° C each for 30 s, followed by a final 2 min at 72 ° C

**Notes.**

* Two primer sets were used to target uninucleated cyst-producing *Entamoeba* species (E Victory, pers. comm., 2010).

Separate PCRs were performed with each primer pair in a reaction mixture of 40 µ l consisting of 4 µ l of extracted DNA, 20 µ l of Biomix (Bioline, UK), 15 µ l of sterile distilled water, and 0.5 µ l of each forward and reverse primer. The amplification reactions were performed using a Bioer Xp Cycler as described in Table 4. PCR products were separated by electrophoresis in 1.2% agarose gels run at 100 V on a Thermo Scientific Easycast B1 or D2 electrophoresis gel tank with a Thermo Scientific EC 1000 XL Power Pac for approximately 60 min. A mix of 7 µ l of PCR product and 3.5 µl of loading buffer (New England Biolabs Ltd., UK) were applied to each well. A 1-kbp molecular size ladder (New England Biolabs) was added to each gel for product size estimation. Gels were stained with 0.1 µ g/ml ethidium bromide solution. Amplified DNA was visualized under UV light.

**Table 4:** Details of purified amplicons of *Entamoeba* species from which nucleotide sequences were obtained.

Primate species	Zoological park	Primers	Target
Banded Leaf Monkey (*Presbytis femoralis*)	Howlett’s Wild Animal Park	RRH3, RRH5	*E. histolytica*
Eastern Javan Langur (*Trachypithecus auratus auratus*)	Twycross Zoo	RRD3, RRD5	*E. dispar*
Dusky Leaf Monkey (*Trachypithecus obscures*)	Twycross Zoo	RRD3, RRD5	*E. dispar*
Dusky Leaf Monkey (*Trachypithecus obscures*)	Howletts Wild Animal Park	RRD3, RRD5	*E. dispar*
Eastern Javan Langur (*Trachypithecus auratus auratus*)	Howletts Wild Animal Park	RRD3, RRD5	*E. dispar*
Eastern Javan Langur (*Trachypithecus auratus auratus*)	Twycross Zoo	RRD3, RRD5	*E. dispar*
Eastern Javan Langur (*Trachypithecus auratus auratus*)	Twycross Zoo	RRD3, RRD5	*E. dispar*
Eastern Javan Langur (*Trachypithecus auratus auratus*)	Twycross Zoo	RRD3, RRD5	*E. dispar*
Eastern Javan Langur (*Trachypithecus auratus auratus*)	Twycross Zoo	RRD3, RRD5	*E. dispar*
Black Howler Monkey (*Alouatta caraya*)	Twycross Zoo	P3.2, P5.2	Uninucleates
Black Howler Monkey (*Alouatta caraya*)	Twycross Zoo	P3.2, P5.2	Uninucleates
Woolly Monkey (*Lagothrix lagotricha*)	Twycross Zoo	P3.2, P5.2	Uninucleates
Golden-headed Lion Tamarin (*Leontopithecus chrysomelas*)	Twycross Zoo	P3.2, P5.2	Uninucleates
Eastern Javan Langur (*Trachypithecus auratus auratus*)	Twycross Zoo	P3.2, P5.2	Uninucleates
Eastern Javan Langur (*Trachypithecus auratus auratus*)	Twycross Zoo	P3.2, P5.2	Uninucleates
Woolly Monkey (*Lagothrix lagotricha*)	Twycross Zoo	P3.2, P5.2	Uninucleates

### Molecular phylogenetics

One positive amplicon per primate (a total of 16 amplicons) was selected for sequencing, based on visualization of PCR products (Table 4). Amplicons were purified, using a QIAquick PCR Purification Kit (QIAgen, UK), according to the manufacturer’s instructions and then subjected to sequencing on the Illumina platform by Source BioScience (Nottingham, UK) using the primers from the PCR. Nucleotide sequences were determined at least once on each DNA strand. Three representative *Entamoeba* nucleotide sequences obtained in this study were deposited in GenBank under accession numbers KJ149294, KJ149295, KJ149296.

Raw sequencing chromatograms were evaluated with Geneious (version 5.4) software. Newly obtained *Entamoeba* sequences were compared with similar sequences available at the GenBank database by using the Bl2Seq algorithm as implemented in BLASTn (Altschul et al., 1990). Multiple alignments of all nucleotide sequences were obtained by using the MUSCLE program (Edgar, 2004). The resulting alignments were adjusted manually when necessary using CLUSTALX (Larkin et al., 2007). The unmatched ends were deleted to obtain a homogeneous matrix of characters and thus increase the reliability of the tree obtained. Phylogenetic trees were inferred from the nucleotide sequence alignments by the maximum-likelihood (ML) method using the BIONJ algorithm (Gascuel, 1997) and distance method with HKY85 model (Hasegawa, Kishino & Yano, 1985) of nucleotide substitution implemented in PhyML-aLRT (Guindon & Gascuel, 2003). The reliability of the branching order was assessed by performing 1,000 bootstrap replicates.

## Results

### *Entamoeba* prevalence at Twycross Zoo

One hundred and twenty-six stool samples were collected from 37 individual primates. No primate demonstrated ill health at the time of sample collection and no samples contained grossly visible blood. Microscopic examination demonstrated *Entamoeba* shedding in 81.1% of 37 primates sampled (Table 1). *Entamoeba coli* was the most prevalent *Entamoeba* species shed (62.2%), with three of six primate species shedding this *Entamoeba* species. Shedding of species from the *E. histolytica* complex was identified in 16.2% of primates (6 primates). Co-infection with two or more *Entamoeba* species was identified in 14 primates. Old World Colobinae primates showed the highest prevalence of *Entamoeba* infection. *Entamoeba* infection was significantly associated with species of primate (*P* < 0.05) and administration of metronidazole (*P* < 0.05). More specifically, infection with *E. coli* was significantly associated with both parameters (both *P* < 0.05). Primates previously treated with metronidazole showed greater infection with *E. coli* (76.9%) compared to those untreated (25.0%). No significant associations were identified between primate demographic characteristics and infection with *Entamoeba* from the *E. histolytica* complex or *E. hartmanni*. Eggs from *Trichuris* species were identified in samples from two primates.

### *Entamoeba* prevalence at multiple zoos

*Entamoeba* was present in 101 (28.9%) samples (Table 5), indicating a notable prevalence of *Entamoeba* infection at the national level. No more than one species of *Entamoeba* was identified per sample. Three *Entamoeba* species were detected by species-specific PCR and confirmed with sequencing and BLAST: *E. histolytica*, *E. dispar* and *E. polecki*. *E. histolytica* was detected in one sample (2.9%), *E. dispar* in 14 samples (4.0%) and uninucleated cyst-producing *Entamoeba* species in 86 (24.6%) samples. *E. histolytica* and *E. dispar* were identified in samples from Colobinae primates only, whilst uninucleated-cyst producing *Entamoeba* species were identified in samples primarily from New World monkeys, but also in primates from the Colobinae family. *Entamoeba* infection was only detected in primates from three zoological parks (Table 5): *E. histolytica* was only identified at one park, *E. dispar* in three parks, and uninucleated cyst-producing *Entamoeba* species in three parks. No primate was found to harbor mixed *Entamoeba* species. All primates sampled appeared clinically healthy at the time of sample collection.

**Table 5:** The prevalence of *Entamoeba* species by species of primate and zoological park.

Species of primate	*E. histolytica*	*E. dispar*	Uninucleates
	% (number of primate)		
**Old world monkey**			
Banded Leaf Monkey (*Presbytis femoralis*; *n* = 20)	5.0 (1)	0	0
Dusky Leaf Monkey (*Trachypithecus obscures*; *n* = 60)	0	3.3 (2)	0
Eastern Javan Langur (*Trachypithecus auratus auratus*; *n* = 117)	0	10.3 (12)	29.1 (34)
Francois Langur (*Trachypithecus francoisi*; *n* = 19)	0	0	0
Grizzled Leaf Monkey (*Presbytis comata*; *n* = 27)	0	0	0
Silvery Langur (*Trachypithecus cristatus cristatus*; *n* = 9)	0	0	0
Purple-faced Langur (*Trachypithecus vetulus vetulus*; *n* = 11)	0	0	0
**Subtotal** (***n*** = **263**)	**0.76 (1)**	**5.3 (14)**	**12.9 (34)**
**New world monkey**			
Black Howler Monkey (*Alouatta caraya*; *n* = 52)	0	0	70.9 (40)
Woolly Monkey (*Lagothrix lagotricha*; *n* = 23)	0	0	47.8 (11)
Golden-headed Lion Tamarin (*Leontopithecus chrysomelas*; *n* = 6)	0	0	16.7 (1)
Golden Lion Tamarin (*Leontopithecus rosalia*; *n* = 6)	0	0	0
**Subtotal** (***n*** = **87**)	**0**	**0**	**59.8 (52)**
**Total** (***n*** = **350**)	**0.3 (1)**	**4.0 (14)**	**24.6 (86)**
**Zoological park**			
Colchester Zoo (*n* = 9)	0	0	0
Cotswold Wildlife Park and Gardens (*n* = 8)	0	0	0
Edinburgh Zoo (*n* = 3)	0	0	0
Howletts Wild Animal Park (*n* = 90)	1.1 (1)	2.2 (2)	0
Port Lympne Wild Animal Park (*n* = 72)	0	2.8 (2)	0
Twycross Zoo (*n* = 168)	0	6.0 (10)	51.2 (86)
**Total** (***n*** = **350**)	**0.3 (1)**	**4.0 (14)**	**24.6 (86)**

**Figure 2:**
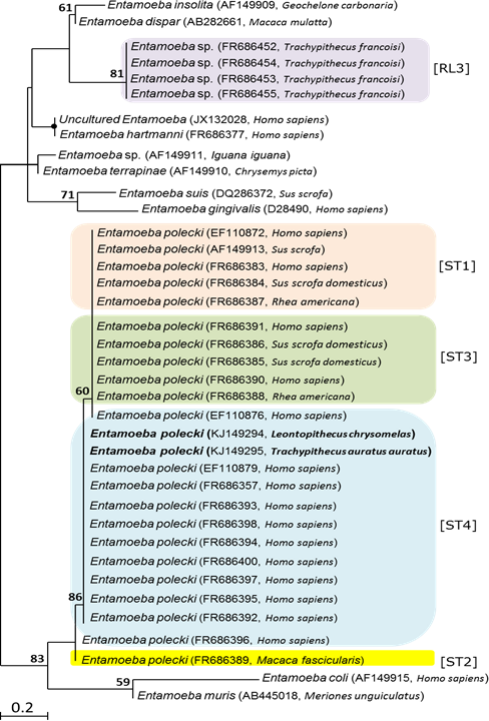
Phylogenetic tree based on partial 18SrDNA sequences, showing the relationships among *Entamoeba* species. Phylogenetic analysis used two different approaches, distance-based analysis and maximum-likelihood (ML), produced trees with identical topologies of which only ML tree is presented. GenBank accession numbers and host species are given in parentheses after the taxon name. Sequences in bold face were obtained during this study. Numbers above branches are bootstrap values (%) from 1,000 replicates. Nodes of the tree with bootstrap values of ≥95% are indicated by black closed circles. The node is not labeled where bootstrap support values is <50. Bar = estimated number of substitutions per site.

To infer the phylogenetic relationship of the isolates detected in the present study, *E. histolytica*, *E. dispar* and *E. polecki*, with previously characterized isolates, we used maximum likelihood method. PCR amplicons from sixteen samples were purified and submitted for sequencing (Table 4). The sequence from the sample that produced an amplicon with the *E. histolytica*-specific primers was identical to the corresponding region of the GenBank sequence for *E. histolytica* from monkey (AB197936) from a cynomolgus monkey. Likewise, sequences obtained from eight samples that produced amplicons with the *E. dispar*-specific primers were identical to the corresponding region of the GenBank sequence (AB282661) for *E. dispar* from a rhesus monkey. Seven sequences were obtained from uninucleate amplicons from Twycross Zoo and shared high sequence homology *E. polecki*. Two representative sequences were used to build a phylogenetic tree. As seen in Fig. 2, *E. polecki* sequences obtained in the present study clustered with and formed a monophyletic group with *E. polecki* subtype 4 isolates reported in *Homo sapiens* from Asia, Africa and Europe.

## Discussion

Nonhuman primates harbour a number of *Entamoeba* spp of varied importance to human and domestic animal health. The prevalence and genetic identity of *Entamoeba* species was investigated in primate collections at six major NHP zoos in the United Kingdom. Results indicated a low prevalence of the pathogenic *E. histolytica* in the examined primates. This is important to the primate population and also to the many thousands of human visitors of these zoos each year. Higher prevalence of non-pathogenic *Entamoeba* species was however identified in the primates sampled. Previous studies utilizing molecular methods to identify carriage of *Entamoeba* species demonstrated a similar prevalence data to that seen in the current study. Low carriage of *E. histolytica* and higher carriage of other *Entamoeba* species in NHP populations has been demonstrated in both captive (Tachibana et al., 2000; Tachibana et al., 2001; Takano et al., 2005; Rivera, Yason & Adao, 2010) and free-living NHP species (Rivera & Kanbara, 1999). However, the NHP populations examined in these studies were based outside of Europe, with all of the captive populations investigated in these studies existing in research facilities in Asia. Levecke et al. (2010) reported that 36% of faecal samples collected from various primate species in zoological parks in Belgium and The Netherlands contained *E. histolytica*, and identified *Entamoeba* species as the most prevalent gastrointestinal parasite within the sampled population.

The lack of sex or age predisposition to infection with *Entamoeba* species in our study is in agreement with other studies (Lilly, Mehlman & Doran, 2002; Jones-Engel et al., 2004; Gillespie, Greiner & Chapman, 2005; Muehlenbein, 2005; Ekanayake et al., 2006; Teichroeb et al., 2009). In the present study, the highest prevalence of *Entamoeba* infection was detected in Old World monkeys; this finding is in agreement with reports from other studies in Japan (Tachibana et al., 2001) and Belgium (Levecke et al., 2007). Primates from the Colobinae family have specialised sacculated stomachs, an adaption to their leaf-eating lifestyle, which provides favorable conditions for ingested *Entamoeba* cyst excystation, and trophozoite tissue invasion (Mätz-Rensing et al., 2004; Ulrich et al., 2010). The higher carriage of uninucleated cyst-producing *Entamoeba* species, compared to other *Entamoeba* species, identified at Twycross Zoo may be explained by the asymptomatic commensal carriage of a non-pathogenic *Entamoeba* species. These non-pathogenic species are less likely to be clinically identified; therefore, infected primates are less likely to receive amoebicidal treatment. Administration of amoebicidal drugs might have been the cause of the apparent increase in the prevalence of uninucleated cyst-producing *Entamoeba* species and *Entamoeba coli* in primates at Twycross Zoo. Re-establishment of gastrointestinal microflora, following treatment with amoebicidal agents, may have favoured the growth of the commensal populations of the octonucleated cyst-producing *Entamoeba* species (*E. coli*) in treated primates, as confirmed by microscopic examination of stool samples. In line with this assumption is the reported high frequency of the commensal uninucleated and octonucleated cyst-producing commensal *Entamoeba* species in primate populations (Tachibana et al., 2000; Petrášová et al., 2010). Alternatively, this may be explained by the development of metronidazole resistance in these uninucleated and octonucleated cyst-producing *Entamoeba* species.

The difference in the prevalence of *Entamoeba* among zoos (Table 5) can be explained by the differences in biosecurity and precautionary measures taken to prevent parasitic disease transmission. All zoos participated in the study already implement routine disinfection programmes (personal communication with Zoos). However, additional precautionary measures are needed in order to prevent the transmission of infection between enclosures including hygienic food preparation, provision of potable water, and disinfection of keeper footwear, over-clothing, hands, and cleaning equipment between enclosures. Effective drainage and water microfiltration within enclosures is also critical. Proactive pest control measures reduce arthropod vectors transporting infective cysts between enclosures (Pang, Chang & Chang, 1993; Denver, 2008). Additionally, avoiding mixed primate exhibits reduces the transmission of amoebiasis between NHP of different susceptibility. The same measure may help to prevent zoonotic transmission to zoo visitors. Unfortunately limited time and financial resources often result in deficiencies in one or more of these measures.

Methods for *Entamoeba* identification have been undergoing rapid change over the past decade and molecular phylogenetic techniques are rapidly becoming the procedures of choice (Levecke et al., 2010; Stensvold et al., 2011). PCR amplification of the 18S rDNA gene directly from a sample of mixed microbiota alleviates the need for culturing *Entamoeba* (Levecke et al., 2010), and once DNA is prepared, there are no biohazard dangers. rDNA-based molecular phylogenetic techniques were used to identify the *Entmaoeba* species detected in the faecal samples from NHP in the present study. Sequences from *E. histolytica* and *E. dispar* obtained in the study were identical to previously reported sequences in Genbank AB197936 and AB282661, respectively. Phylogenetic analysis of the partial-length 18SrDNA sequence showed that the uninucleate amplicons from Twycross Zoo were all *E. polecki*.

The uninucleated-cyst-producing *Entamoeba* infecting humans *E. polecki* species complex has been found to encompass four subtypes (ST1–ST4) (Stensvold et al., 2011). *E. polecki* ST1 (previously given to *E. polecki* in pigs); ST2 (*E. chattoni* from non-human primates); ST3 (*E. struthionis* from pigs and ostriches); and ST4 (restricted to humans; unlikely to be zoonotic); indicating low host specificity of ST1 and ST3. Comparison between sequences obtained in the present study and reference sequences obtained from GenBank for each of the four *E. polecki* subtypes indicated that sequences of *E. polecki* obtained in the present study from NHPs [Woolly Monkey (*Lagothrix lagotricha*), Eastern Javan Langur (*Trachypithecus auratus auratus*), Golden-headed Lion Tamarin (*Leontopithecus chrysomelas*), and Black Howler Monkey (*Alouatta caraya*)] formed a phylogenetic cluster (Fig. 2) with isolates of *E. polecki* subtype 4 reported in *Homo sapiens* from Africa, Asia and Europe (Stensvold et al., 2011). Given the reported high specificity of *E. polecki* subtype 4 to humans, the similarity between sequences obtained from NHPs from Twycross zoo in the present study with *E. polecki* ST4 sequences obtained from *Homo sapiens* suggest a zoonotic potential. However, more analysis is needed before any suggestion about the zoonotic implication of the isolates obtained in this study to be made. A group of uninucleate Entamoebas (referred to as *Entamoeba* RL3), phylogenetically distant from the *E. polecki* complex, have been reported from Francois Langur (*Trachypithecus francoisi*) from Twycross Zoo in England (Stensvold et al., 2011). Interestingly, sequences of this *Entamoeba* RL3 group did not seem to share similarity with the sequences of uninucleated-cyst-producing *E. polecki* obtained in the present study from NHPs from the same Zoo.

Sequence data (Table 4) also suggest that a common source asymptomatic infection with the uninucleated cyst-producing *Entamoeba* species, *E. polecki*, at Twycross Zoo may have propagated through many primate enclosures. This study did not examine the prevalence of *E. nutalli*, an emerging species currently seeming to be prevalent in NHPs (Tachibana et al., 2013). Since *E. nuttalli* has been associated with symptomatic carriage, and appears to be restricted in host distribution to NHPs, it would be interesting to know whether any of the animals sampled in the present study harboured *E. nuttalli*. Thus, further studies are needed to establish the prevalence of this important species in NHPs in the United Kingdom and its zoonotic risk to public health.

## Conclusion

This is the first study to report the prevalence of *Entamoeba* infection in captive NHPs in the United Kingdom. Data collected from six zoos suggests a notable prevalence of *Entamoeba* infection in NHPs in UK. DNA sequencing of positive stool samples revealed three main species of *Entamoeba*, *E. histolytica*, *E. dispar* and *E. polecki* ST4 circulating in the zoo’s environment in the UK. Some *Entamoeba* species can have zoonotic potential, thus can constitute a risk for humans who are in close contact with primates.

## Supplemental Information

10.7717/peerj.492/supp-1 Supplemental Information Raw Data

## Additional Information and Declarations

### DNA Deposition

The following information was supplied regarding the deposition of DNA sequences: GenBank KJ149294, KJ149295, KJ149296.

### Data Deposition

The following information was supplied regarding the deposition of related data: Nottingham ePrints Archive (http://eprints.nottingham.ac.uk/).

## References

[ref-1] Ali IKM, Zaki M, Clark CG (2005). Use of PCR amplification of tRNA gene-linked short tandem repeats for genotyping *Entamoeba histolytica*. Journal of Clinical Microbiology.

[ref-2] Altschul SF, Gish W, Miller W, Myers EW, Lipman DJ (1990). Basic local alignment search tool. Journal of Molecular Biology.

[ref-3] Denver MC, Fowler ME, Miller RE (2008). Reptile protozoa. Zoo and wild animal medicine current therapy.

[ref-4] Edgar RC (2004). MUSCLE: multiple sequence alignment with high accuracy and high throughput. Nucleic Acids Research.

[ref-5] Ekanayake DK, Arulkanthan A, Horadagoda NU, Sanjeevani GKM, Kieft R, Gunatilake S, Dittus WPJ (2006). Prevalence of *crytosporidium* and other enteric parasites among wild non-human parasites in Polonnaruwa, Sri Lanka. American Journal of Tropical Medicine and Hygiene.

[ref-6] Feng M, Yang B, Yang L, Fu Y, Zhuang Y, Liang L, Xu Q, Cheng X, Tachibana H (2011). High prevalence of *Entamoeba* infections in captive long-tailed macaques in China. Parasitology Research.

[ref-7] Fotedar R, Stark D, Beebe N, Marriott D, Ellis J, Harkness J (2007). Laboratory diagnostic techniques for *Entamoeba* species. Clinical Microbiology Reviews.

[ref-8] Gascuel O (1997). BIONJ: an improved version of the NJ algorithm based on a simple model of sequence data. Molecular Biology and Evolution.

[ref-9] Gillespie TR, Greiner EC, Chapman CA (2005). Gastrointestinal parasites of the Colobus Monkey of Uganda. Journal of Parasitology.

[ref-10] Guindon S, Gascuel O (2003). A simple, fast, and accurate algorithm to estimate large phylogenies by maximum likelihood. Systematic Biology.

[ref-11] Hasegawa M, Kishino H, Yano T (1985). Dating of the human-ape splitting by a molecular clock of mitochondrial DNA. Journal of Molecular Evolution.

[ref-12] Heredia RD, Fonseca JA, Lopez MC (2012). *Entamoeba moshkovskii* perspectives of a new agent to be considered in the diagnosis of amebiasis. ACTA Tropica.

[ref-13] Jones-Engel L, Engel GA, Schillaci MA, Kyes K, Froehlich J, Paputungan U, Kyes RC (2004). Prevalence of enteric parasites in pet macaques in Sulawesi, Indonesia. American Journal of Primatology.

[ref-14] Kebede A, Verweij J, Dorigo-Zetsma W, Sanders E, Messele T, van Lieshout L, Petros B, Polderman T (2003). Overdiagnosis of amoebiasis in the absence of *Entamoeba histolytica* among patients presenting with diarrhoea in Wonji and Akaki, Ethiopia. Transactions of the Royal Society of Tropical Medicine and Hygiene.

[ref-15] Larkin MA, Blackshields G, Brown NP, Chenna R, McGettigan PA, McWilliam H, Valentin F, Wallace IM, Wilm A, Lopez R, Thompson JD, Gibson TJ, Higgins DG (2007). Clustal W and clustal X version 2.0. Bioinformatics.

[ref-16] Levecke B, Dorny P, Geurden G, Vercammen F, Vercruysse J (2007). Gastrointestinal protozoa in non-human primates of four zoological gardens in Belgium. Veterinary Parasitology.

[ref-17] Levecke B, Dreesen L, Dorny P, Verweij JJ, Vercammen F, Casaert S, Vercruysse J, Geldhof P (2010). Molecular identification of *Entamoeba* spp. in captive nonhuman primates. Journal of Clinical Microbiology.

[ref-18] Lilly AA, Mehlman PT, Doran D (2002). Intestinal parasites in gorillas, chimpanzees, and humans at Mondika Research Site, Dzanga-Ndoki National Park, Central African Republic. International Journal of Primatology.

[ref-19] Mätz-Rensing K, Floto A, Kaup FJ, Schaller K (2004). Gastric amoebiasis due to *Entamoeba histolytica* infection a mandrill (*Mandrillus sphinx*).

[ref-20] Muehlenbein MP (2005). Parasitological analyses of the male chimpanzees (*Pan troglodytes schweinfurthii*) at Ngogo, Kibale National Park, Uganda. American Journal of Primatology.

[ref-21] Pang VF, Chang CC, Chang WF (1993). Concurrent gastric and hepatic amebiasis in a dusky leaf monkey (*Presbytis obscurus*). Journal of Zoo and Wildlife Medicine.

[ref-22] Petrášová J, Modrý D, Huffman MA, Mapua MI, Bobáková L, Mazoch V, Singh J, Kaur T, Petrželková KJ (2010). Gastrointestinal parasites of indigenous and introduced primate species of Rubondo Island National Park, Tanzania. International Journal of Primatology.

[ref-23] Rivera WL, Kanbara H (1999). Detection of *Entamoeba dispar* DNA in macaque feces by polymerase chain reaction. Parasitology Research.

[ref-24] Rivera WL, Yason JADL, Adao DEV (2010). *Entamoeba histolytica* and *E. dispar* infections in captive macaques (*Macaca fascicularis*) in the Philippines. Primates.

[ref-25] Sargeaunt PG, Patrick S, O’Keeffe D (1992). Human infections of *Entamoeba chattoni* masquerade as *Entamoeba histolytica*. Transactions of the Royal Society of Tropical Medicine and Hygiene.

[ref-26] Sargeaunt PG, Williams JE, Jones DM (1982). Electrophoretic isoenzyme patterns of *Entamoeba histolytica* and *Entamoeba chattoni* in a primate survey. Journal of Protozoology.

[ref-27] Solaymani-Mohammadi S, Rezaian M, Babaei Z, Rajabpour A, Meamar AR, Pourbabai AA, Petri WA (2006). Comparison of a stool antigen detection kit and PCR for diagnosis of *Entamoeba histolytica* and *Entamoeba dispar* infections in asymptomatic cyst passers in Iran. Journal of Clinical Microbiology.

[ref-28] Stensvold CR, Lebbad M, Victory EL, Verweij JJ, Tannich E, Alfellani M, Legarraga P, Clark CG (2011). Increased sampling reveals novel lineages of *Entamoeba*: consequences of genetic diversity and host specificity for taxonomy and molecular detection. Protist.

[ref-29] Tachibana H, Cheng XJ, Kobayashi S, Fujita Y, Udono T (2000). *Entamoeba dispar*, but not *E. histolytica*, detected in chimps. Parasitology Research.

[ref-30] Tachibana H, Cheng XJ, Kobayashi S, Matsubayashi N, Gotoh S, Matsubayashi K (2001). High prevalence of infection with *Entamoeba dispar*, but not *E. histolytica*, in captive macaques. Parasitology Research.

[ref-31] Tachibana H, Yanagi T, Lama C, Pandey K, Feng M, Kobayashi S, Sherchand JB (2013). Prevalence of *Entamoeba nuttalli* infection in wild rhesus macaques in Nepal and characterization of the parasite isolates. Parasitology International.

[ref-32] Takano J, Narita T, Tachibana H, Shimizu T, Komatsubara H, Terao K, Fujimoto K (2005). *Entamoeba histolytica* and *Entamoeba dispar* infections in cynomolgus monkeys imported into Japan for research. Parasitology Research.

[ref-33] Teichroeb JA, Kutz SJ, Parkar U, Thompson RCA, Sicotte P (2009). Ecology of the gastrointestinal parasites of *Colobus vellerosus* at Boabeng-Fiema, Ghana: possible anthropozoonotic transmission. American Journal of Physical Anthropology.

[ref-34] Ulrich R, Böer M, Herder V, Spitzbarth I, Hewicker-Trautwein M, Baumgärtner W, Wohlsein P (2010). Epizootic fatal amebiasis in an outdoor group of Old World monkeys. Journal of Medical Primatology.

[ref-35] Verweij JJ, Vermeer J, Brienen EAT, Blotkamp C, Laeijendecker D, van Lieshout L, Polderman AM (2003). *Entamoeba histolytica* infections in captive primates. Parasitology Research.

[ref-36] World Health Organization (1997). Amoebiasis. Weekly Epidemiology Record.

