# Supplementary material for: Prevalence of Entamoeba species in captive primates in zoological gardens in the UK
Source: PeerJ. 2014 Jul 29;2:e492. doi: 10.7717/peerj.492 (PMC4121542; doi:10.7717/peerj.492)
Supplement: Supplemental Information [file peerj-02-492-s001.docx]

GenBank flat file:

LOCUS KJ149294 601 bp DNA linear INV 12-MAY-2014

DEFINITION Entamoeba chattoni isolate WOSMR1 small subunit ribosomal RNA gene,

partial sequence.

ACCESSION KJ149294

VERSION KJ149294

KEYWORDS .

SOURCE Entamoeba chattoni

ORGANISM Entamoeba chattoni

Eukaryota; Amoebozoa; Archamoebae; Entamoebidae; Entamoeba.

REFERENCE 1 (bases 1 to 601)

AUTHORS Elsheikha,H.M.

TITLE Entamoeba species in captive primates in zoological gardens in the

UK

JOURNAL Unpublished

REFERENCE 2 (bases 1 to 601)

AUTHORS Elsheikha,H.M.

TITLE Direct Submission

JOURNAL Submitted (09-JAN-2014) Veterinary Medicine and Science, University

of Nottingham, College Road, Loughborough, Leicestershire LE12 5RD,

United Kingdom

COMMENT ##Assembly-Data-START##

Assembly Method :: Geneious Pro v. 5.4.6

Sequencing Technology :: Illumina

##Assembly-Data-END##

FEATURES Location/Qualifiers

source 1..601

/organism="Entamoeba chattoni"

/mol_type="genomic DNA"

/isolate="WOSMR1"

/isolation_source="host feces"

/host="Leontopithecus chrysomelas (golden headed lion

tamarin)"

/db_xref="taxon:110765"

/country="United Kingdom"

/collection_date="17-Aug-2010"

/collected_by="Carl Regan"

/identified_by="Hany Elsheikha"

rRNA <1..>601

/product="small subunit ribosomal RNA"

ORIGIN

1 atagcttttt gagaagaagg ttaaattgag aattgatgaa acttaaatgt ttttacaagt

61 aactgtttta aatatctgac ctatcaactt gaaggtatga tagaggcata cccaagtgat

121 aacgggtaac gagaaataag ggtttgattt cggagaggga gctttaaaaa tggctaccac

181 ttctaaggaa ggcagcaggc gcgcaaatta cccactttta atttagagag gtagtgacga

241 taattaatag gatttgtttt ataacaagtt caattgtaat gattagagtg taaaaaaatt

301 taagaaatcg attggagggc aagtctggtg ccagcagccg cggtaattcc agctccaata

361 gtgtatatta aagttgttgt gtttaaaaag ctcgtagtcg aattataggg tttttatgtt

421 agtaattcta ttactaggat agaaaaagga gaaaggttat ttataatctt ttatttactt

481 tgaaaaaaat agagtgttca aagcaaaagt caattaatgt ataatgaagc ataggataat

541 aatgaggaga agaaattttt gagaacagta ttaaaaaggg aaattggggt aatttagaca

601 a

//

LOCUS KJ149295 604 bp DNA linear INV 12-MAY-2014

DEFINITION Entamoeba chattoni isolate WOSMR2 small subunit ribosomal RNA gene,

partial sequence.

ACCESSION KJ149295

VERSION KJ149295

KEYWORDS .

SOURCE Entamoeba chattoni

ORGANISM Entamoeba chattoni

Eukaryota; Amoebozoa; Archamoebae; Entamoebidae; Entamoeba.

REFERENCE 1 (bases 1 to 604)

AUTHORS Elsheikha,H.M.

TITLE Entamoeba species in captive primates in zoological gardens in the

UK

JOURNAL Unpublished

REFERENCE 2 (bases 1 to 604)

AUTHORS Elsheikha,H.M.

TITLE Direct Submission

JOURNAL Submitted (09-JAN-2014) Veterinary Medicine and Science, University

of Nottingham, College Road, Loughborough, Leicestershire LE12 5RD,

United Kingdom

COMMENT ##Assembly-Data-START##

Assembly Method :: Geneious Pro v. 5.4.6

Sequencing Technology :: Illumina

##Assembly-Data-END##

FEATURES Location/Qualifiers

source 1..604

/organism="Entamoeba chattoni"

/mol_type="genomic DNA"

/isolate="WOSMR2"

/isolation_source="host feces"

/host="Trachypithecus auratus auratus (Eastern Javan

langur)"

/db_xref="taxon:110765"

/country="United Kingdom"

/collection_date="28-Aug-2010"

/collected_by="Carl Regan"

/identified_by="Hany Elsheikha"

rRNA <1..>604

/product="small subunit ribosomal RNA"

ORIGIN

1 gaatagcctt tttgagaaga aggttaaatt gagaattgat gaaacttaaa tgtttttaca

61 agtaactgtt ttaaatatct gacctatcaa cttgaaggta tgatagaggc atacccaagt

121 gataacgggt aacgagaaat aagggtttga tttcggagag ggagctttaa aaatggctac

181 cacttctaag gaaggcagca ggcgcgcaaa ttacccactt ttaatttaga gaggtagtga

241 cgataattaa taggatttgt tttataacaa gttcaattgt aatgattaga gtgtaaaaaa

301 atttaagaaa tcgattggag ggcaagtctg gtgccagcag ccgcggtaat tccagctcca

361 atagtgtata ttaaagttgt tgtgtttaaa aagctcgtag tcgaattata gggtttttat

421 gttagtaatt ctattactag gatagaaaaa ggagaaaggt tatttataat cttttattta

481 ctttgaaaaa aatagagtgt tcaaagcaaa agtcaattaa tgtataaaga agcataggat

541 aataatgagg agaagaaatt tttgagaaca gtattaaaaa gggaaattgg ggtaatttag

601 acaa

//

LOCUS KJ149296 900 bp DNA linear INV 12-MAY-2014

DEFINITION Entamoeba dispar isolate PRIR1 trnR-trnR intergenic spacer, partial

sequence.

ACCESSION KJ149296

VERSION KJ149296

KEYWORDS .

SOURCE Entamoeba dispar

ORGANISM Entamoeba dispar

Eukaryota; Amoebozoa; Archamoebae; Entamoebidae; Entamoeba.

REFERENCE 1 (bases 1 to 900)

AUTHORS Elsheikha,H.M.

TITLE Entamoeba species in captive primates in zoological gardens in the

UK

JOURNAL Unpublished

REFERENCE 2 (bases 1 to 900)

AUTHORS Elsheikha,H.M.

TITLE Direct Submission

JOURNAL Submitted (09-JAN-2014) Veterinary Medicine and Science, University

of Nottingham, College Road, Loughborough, Leicestershire LE12 5RD,

United Kingdom

COMMENT ##Assembly-Data-START##

Assembly Method :: Geneious Pro v. 5.4.6

Sequencing Technology :: Illumina

##Assembly-Data-END##

FEATURES Location/Qualifiers

source 1..900

/organism="Entamoeba dispar"

/mol_type="genomic DNA"

/isolate="PRIR1"

/isolation_source="feces"

/host="Trachypithecus auratus auratus (Eastern Javan

langur)"

/db_xref="taxon:46681"

/country="United Kingdom"

/collection_date="09-May-2011"

/collected_by="Carl Regan"

/identified_by="Hany Elsheikha"

misc_feature <1..>900

/note="trnR-trnR intergenic spacer"

ORIGIN

1 catatataga tctgtcttag ataaaatcat tatatttcat gagacaccta tcgtttagca

61 tatcttttat ttttcggtct tttatatgtc tgtttctctg agagacaaag gcaatacctc

121 actatgcaaa aagtcgactg gaggtccatc ttccgactgc ataataatag tatagtcgta

181 tatccctata atttacaatt tccatactcc aatattacaa tctgcttaaa ttcgcaattt

241 aagagagcag aggcgtgaat gttgaaaatt atgaggcgcc tttttatcat cggcactgtc

301 ggtgcggcaa tatatctaaa tcccctatac ggacttatgc tgtacataat acatatcatt

361 tcatctgtta tagtaggaat attattccga ttttacggca aaaaccgcca caactcaccg

421 cccacaaaca taaacaccgc agagcttgaa ctgccacgtg ttattactac agctcttgaa

481 accgcatcaa aaaatatatt aaccgtctgt ttttcgagtg tgttcaatat catcatcccc

541 tatttgagca gcttcctata ctaaattcgc ccttgattga aatattttca gatccactaa

601 aatattctcc gtgcttgtaa atgatgcatg ttcgctcgtg aaatatgtaa ccccaccctt

661 tgagtgttcc cactcgtata actcctatta tttatgttca tatttttgaa ttaatttagt

721 cgaatatttt ataaaattta ctatcccaga aagtgtgcta atatgttcat cctatataat

781 catattaggt catcacgtta ttgtgttatt aggatattat tctataatga atgaatgtgt

841 cttatatcat tatcccctaa tgttattatg ttcttatgag tgtgttcaat atcatcatcc //
